# Asthma, allergies and respiratory symptoms in different activity groups of swimmers exercising in swimming halls

**DOI:** 10.1186/s13102-021-00349-2

**Published:** 2021-10-04

**Authors:** Marja Päivinen, Kari Keskinen, Tuula Putus, Urho M. Kujala, Pentti Kalliokoski, Heikki O. Tikkanen

**Affiliations:** 1 Department of Occupational Medicine, Faculty of Medicine, University of Turku, Turku, Finland; 2 Institute of Clinical Medicine, Faculty of Medicine, University of Helsinki, Helsinki, Finland; 3 Faculty of Sport and Health Sciences, University of Jyväskylä, Jyväskylä, Finland; 4 Department of Environmental and Biological Sciences, University of Eastern Finland, Kuopio, Finland; 5 School of Medicine, Institute of Biomedicine/Sports and Exercise Medicine, University of Eastern Finland, Kuopio, Finland

**Keywords:** Asthma, Allergy, Respiratory symptoms, Pulmonary function, Swimming halls, Swimming

## Abstract

**Background:**

Respiratory symptoms are common in competitive swimmers. However, among these and in swimmers at other activity levels the swimming distance, the total spent time in swimming halls and their medical background varies. Our objectives were, first, to assess their medical histories and the associations with respiratory symptoms among swimmers in different activity groups and then second, to study the pulmonary function findings and related medications in competitive swimmers who exercise in swimming hall environments the most.

**Methods:**

First, 1118 participants consisting of 133 competitive-, 734 fitness- and 251 occasional swimmers answered questionnaires concerning their medical background, their respiratory symptoms in connection to swimming distance and their amount of time spent in swimming halls. Secondly, in 130 competitive swimmers, pulmonary function was tested by spirometry and a specific questionnaire was used to assess respiratory symptoms, medical histories and prescribed medication.

**Results:**

Respiratory symptoms were reported by 18% of the studied swimmers. Competitive swimmers had significantly more symptoms than fitness- and occasional swimmers. Naturally competitive swimmers swum more than 2000 m and stayed by the pool more than 90 min, longer than the other activity groups of swimmers. Spirometry testing showed airway obstruction in 15 swimmers, which was 12% of the 130 competitive swimmers. 21 of them, had physician-diagnosed asthma and 16 of these individuals had prescribed medication for it.

**Conclusions:**

Competitive swimmers had the highest swimming hall exposure and reported significantly more respiratory symptoms. A high prevalence of airway obstruction findings in competitive swimmers with asthma and allergies suggests a need for future recommendations for regular testing and special medical care for competitive swimmers.

## Background

A swimming hall environment, and especially exposure to airborne trichloroamine and related adverse effects on respiratory health, have been intensively investigated in several studies [1–9]. Swimming is recommended as a suitable mode of exercise for persons with asthma and allergies [10–16]].

Swimming induces less respiratory symptoms than other endurance modes of exercise like running or cycling at the same exercise intensity [10–12, 17, 18]. However, among competitive swimmers, the prevalence of respiratory symptoms, such as wheezing, shortness of breath, coughing and mucus production from airways, is reported higher than in the general population [3, 8, 19–23]. Thus, Dropnik et al. [20] suggested, that competitive swimmers with higher intensities and training volumes are exposed to a swimming hall environment more in comparison to those with lower swimming intensities and shorter stays in a swimming pool area. In a study of competitive swimmers, however, the exposure to a swimming hall environment during training years or in weekly training sessions did not associate with respiratory symptoms [21]. In addition, previously the airborne trichloroamine levels in studied swimming halls were low and well below the proposed standard [24]. Therefore, chemical exposure may not be the only explanation and underlying factor for reported respiratory symptoms in connection to swimming. Understanding the role of other factors may require more investigation.

A swimmer’s medical history such as physician-diagnosed asthma, allergies and previous respiratory infection, was a significant underlying factor for reported respiratory symptoms in competitive swimmers [21]. However, studies of the reported symptoms and medical histories among other swimmer activity groups, who use swimming halls, are lacking. Therefore, the aim of this study was to investigate asthma, allergies and respiratory symptoms together with the related exposure to a swimming hall environment in three activity groups and to study further the reported respiratory symptoms, medical histories, pulmonary function and prescribed medication in highly, trained active competitive swimmers.

## Methods

### Subjects

The study subjects using swimming halls were divided into three different groups according to their physical activity level by self-assessment. First, 1118 study participants consisting of competitive-, fitness- and occasional swimmers were examined with a modified structured questionnaire. Then, the training frequency, swimming distance and stay at a swimming pool area were also asked (Table 1).

**Table 1:** Summary of methods and demographics of the study target groups

Study method	Swimmer population, N	Gender F/M	Mean age (yr)	Distance swam per session (meters)	Stay at swimming pool area (minutes)	Asthma Prevalence %	Allergy Prevalence %	Respiratory symptoms Prevalence %
Questionnaire	1118 Swimmers: 251 Occasional 734 Fitness 133 Competitive	650/468 169/82 426/308 55/78	43 (19) 39 (19) 48 (16) 20 (12)	1215 543 993 3418	63 60 56 117	9 12 8 13	23 23 22 28	18 13 16 44
Spirometry	130 Competitive	58 /72	17 (3)	5600	120	20	31	45

Secondly, 130 competitive swimmers participated in both pulmonary function testing and questionnaire surveys. Pulmonary function by spirometry was performed in 130 active and competitive swimmers. Each swimmer was a member of a competitive swimming training team and had a significant swimming training history for several years.

### Questionnaire

Swimming hall users (N = 1118) were asked to participate in the study by filling a structured questionnaire modified from previously used questionnaire [21, 25]. The questionnaire included questions concerning medical history, swimming background and reporting of respiratory symptoms. The medical history of physician-diagnosed asthma and allergies was documented. The swimming distance in meters, time spent at the pool area and number of weekly training sessions were asked. The questionnaires were collected at five different swimming halls located in different parts of Finland.

### Collection of data

Questionnaire data for the swimming hall customer study were collected in swimming halls, suchthat every swimmer could participate in the study within a three-week periodtaking place in the autumn. Autumn allowed for the for any exclusion possible effects of a pollen season on the reporting of respiratory symptoms. There was no information about the exact number of customers during those days in the swimming halls, so the exact participation rates could not be calculated. All swimmers, who participated in pulmonary function testing, filled the questionnaire in connection to the pulmonary function testing.

A specially modified questionnaire was used for the 130 competitive swimmers who participated in spirometry testing (Table 1). All competitive swimmers participated in national swimming championships and had a training history of competitive swimming for 8 years on average. The questionnaire included further questions concerning medical family history and prevalence of respiratory symptoms at different swimming training intensities. The intensities were categorized into five different training zones. Those intensities are known among competitive swimmers, because they are taught and monitored by coaches during training sessions.

### Pulmonary function testing

Highly trained competitive swimmers (N = 130; 58 females and 72 males) participated in both pulmonary function testing and the questionnaire survey. The mean age of the swimmers was 17 years (SD ± 3 years). All of the studied swimmers were participants of national swimming championships with a training history of competitive swimming for 8 years and weekly training sessions of 18 h on average.

Spirometry was measured with a Spiro Star 2000 spirometer (Medikro, Kuopio, Finland) according to the ATS and ERS guidelines [26, 27]. Spirometry results were expressed as a percent of personal age and size- and sex-matched predicted values [28] or for Finnish children under 18 years [29], according to the most recent ATS and ERS criteria. Forced vital capacity (FVC) and forced expiratory volume in one second (FEV_1_) and their ratio (FEV_1_/FVC) were analyzed. Airway obstruction was defined by the criteria used in relation to the age, size and sex matched predicted values. Airway obstruction required a finding of FEV_1_/FVC < 88% of predicted. All studied competitive swimmers were non-smokers.

### Medication prescribed for the swimmers

The medication data were collected and classified by the ATC index from all swimmers participating in spirometry testing.

### Ethics

The ethical considerations for the questionnaire and pulmonary function testing on land were evaluated bythe ethics Committee of Helsinki and Uusimaa hospital district.

### Statistical methods

Odds ratios (OR) together with confidence intervals for reported respiratory symptoms between different risk factor categories were calculated by multiple logistic regression analysis. In this study, the outcome was reported as respiratory symptoms or no respiratory symptoms. Explanatory variables in the model were the activity group (i.e., competitive, fitness or occasional), type of swimming hall, sex and age. In separate models, swimming distance and time spent in the pool area were used. However, in these models the activity group was naturally confounded and a stronger predictor. Therefore, we ran also separate models using swimming distance and the time spent in a swimming hall as well as the type of swimming hall, sex and age but not the activity group. Different cutoffs were assessed, and the greater than 90-min or less than 90-min cutoffs were optimal for time spent. Similarly, for distance, an optimal cutoff of less than 2000 m and greater than 2000 m was used. A P value of less than 0.05 (two tailed) was considered as statistically significant. The data analysis for this study was generated by using SAS software, version 9.4 of the SAS system for Windows (SAS Institute Inc., Gary, NC, USA).

## Results

The prevalence of respiratory symptoms, while swimming, was 18%, when all three activity groups were observed together. The prevalence of physician-diagnosed asthma and allergy was 7%, only asthma was 2% and only allergy was 16%. Neither asthma nor allergy was reported by 836 or 75% of the swimming hall swimmers. The medical histories of physician-diagnosed asthma, allergy and reported respiratory symptoms in different populations of swimmers in swimming halls are shown in Table 1.

**Fig. 1:**
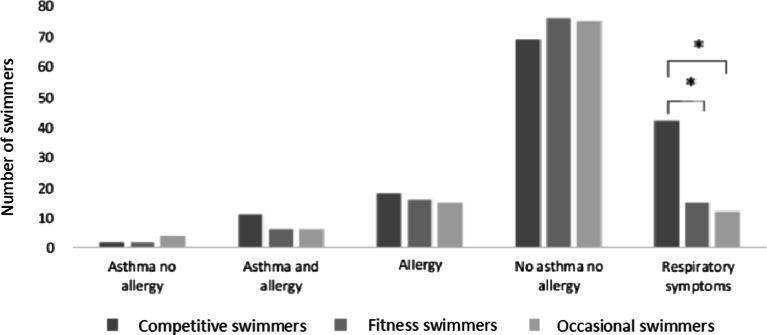
Prevalence of reported respiratory symptoms and physician-diagnosed asthma and allergy in competitive-, fitness- and occasional swimmers

A comparison among different groups of swimmers showed that competitive swimmers reported significantly more respiratory symptoms while swimming than fitness and occasional swimmers (Fig. 1). OR for reporting respiratory symptoms in competitive swimmers were 2.31 (95% CI 1.41–3.80, *P* < 0.0001) compared to fitness swimmers and 5.27 (95% CI 3.19–8.69, *P* < 0.0001) compared to occasional swimmers (Fig. 1).

**Fig. 2:**
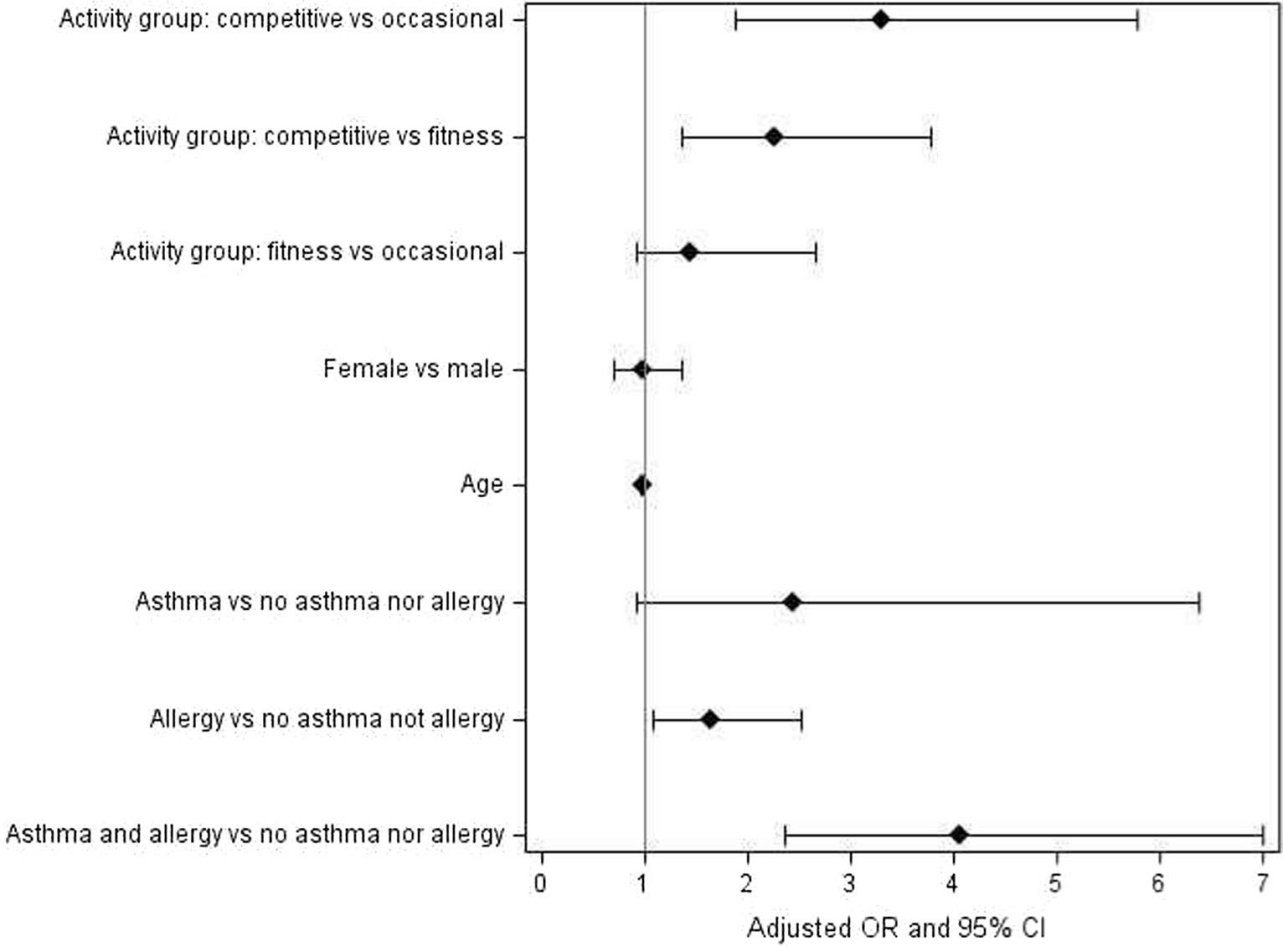
Odds ratio (OR) for respiratory symptoms when activity group, sex, age and medical histories were observed

The highest risk for reported symptoms was observed by the activity group competitive swimmers with a medical history of asthma and allergy. The calculations showed that there were no significant differences between different swimming halls, age or sex (Fig. 2).

Among those with asthma risk for reported respiratory symptoms, they did not differ between different activity groups. However, the risk for reported respiratory symptoms was significantly higher in competitive swimmers who reported only allergy (*P* = 0.001) or neither asthma nor allergy (*P* < 0.0001) in comparison to other swimming hall users, fitness swimmers and occasional swimmers.

**Fig. 3:**
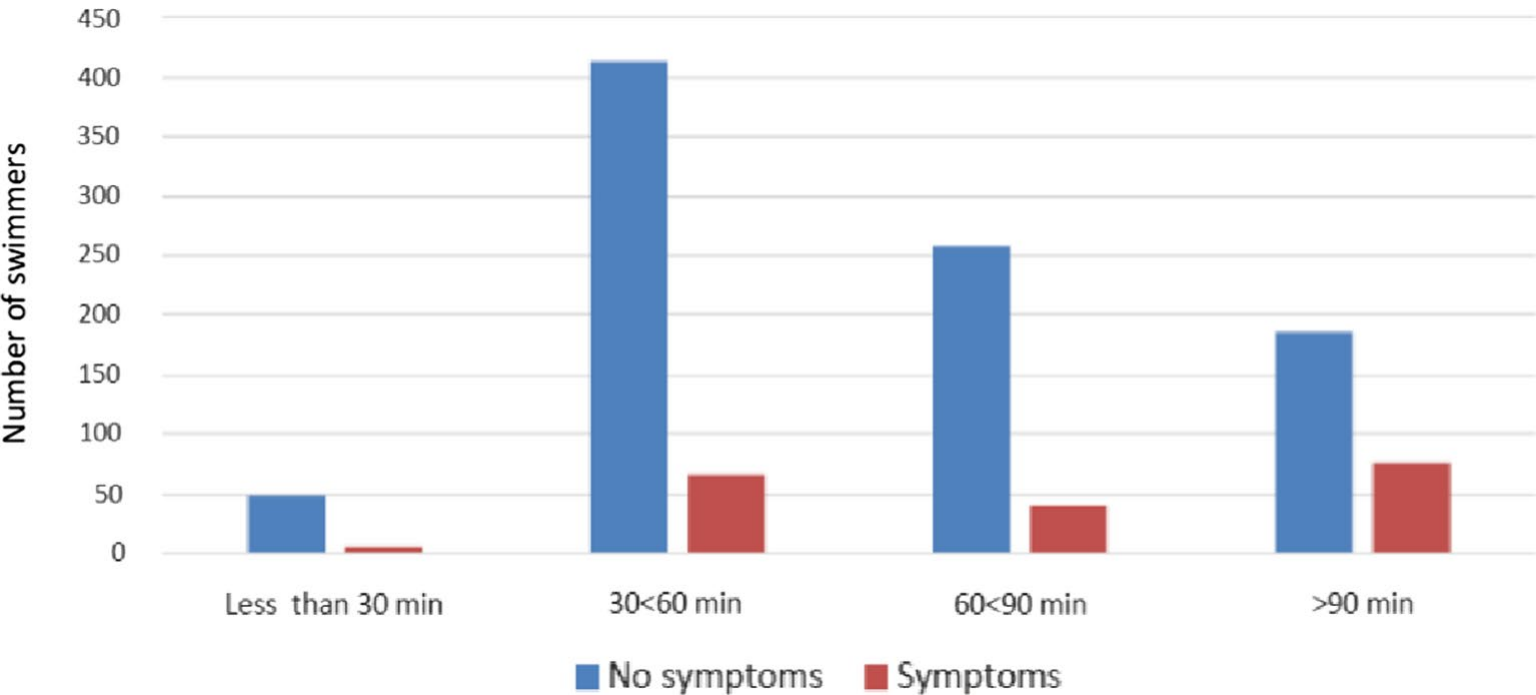
Reported respiratory symptoms by swimming hall users according to the time spent at the swimming pool area was less than 30 min, 30–60 min, 60–90 min or greater 90 min

Examinations on the exposure to a swimming hall environment showed no association between reported symptoms and time spent at the pool area, when the time was less than 90 min. However, the swimmers who spent time in a swimming pool area for more than 90 min reported significantly more respiratory symptoms (OR 2.66, 95% CI1.91–−3.71, *P* < 0.0001) (Fig. 3). This result, however, is strongly associated with the activity group and the fact that the competitive swimmers spent approximately a mean of two hours in the pool area per swimming session, when fitness- and occasional swimmers spent approximately a mean of one hour (Table 1). When additional analyses without the activity group was performed, the time spent factor (< 90 min, ≥ 90 min) was significant (*P* = 0.027) when adjusted with factors of swimming hall type, sex and age.

**Fig. 4:**
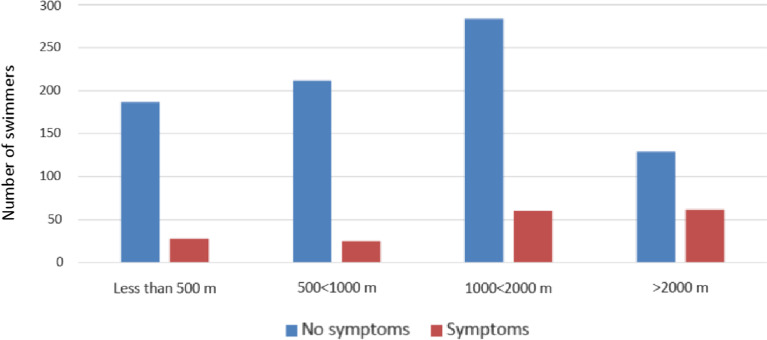
Reported respiratory symptoms in swimming hall users according to a swimming distance of less than 500 m, greater than 500 m but less than 1000, greater than 1000 m, greater than 1000 m but less than 2000 m or greater than 2000 m

There was no association with reported symptoms and distance swum in meters even with less than 2000 m swum. However, the swimmers, who swam more than 2000 m, reported respiratory symptoms significantly more (OR 2.91, 95% CI 2.02–4.17, *P* < 0.0001) compared to the others (Fig. 4). These swimmers were mainly competitive swimmers. This result, however, was also strongly associated with the activity group. The competitive swimmers swum approximately a mean of 3.5 km, when fitness swimmers swum approximately 1 km and occasional swimmers swum approximately 0.5 km per swimming session (Table 1). When additional analyses without the activity group was performed, the distance factor (< 2000 m, ≥ 2000 m) was significant (*P* = 0.0041) when adjusted with swimming hall type, sex and age.

**Fig. 5:**
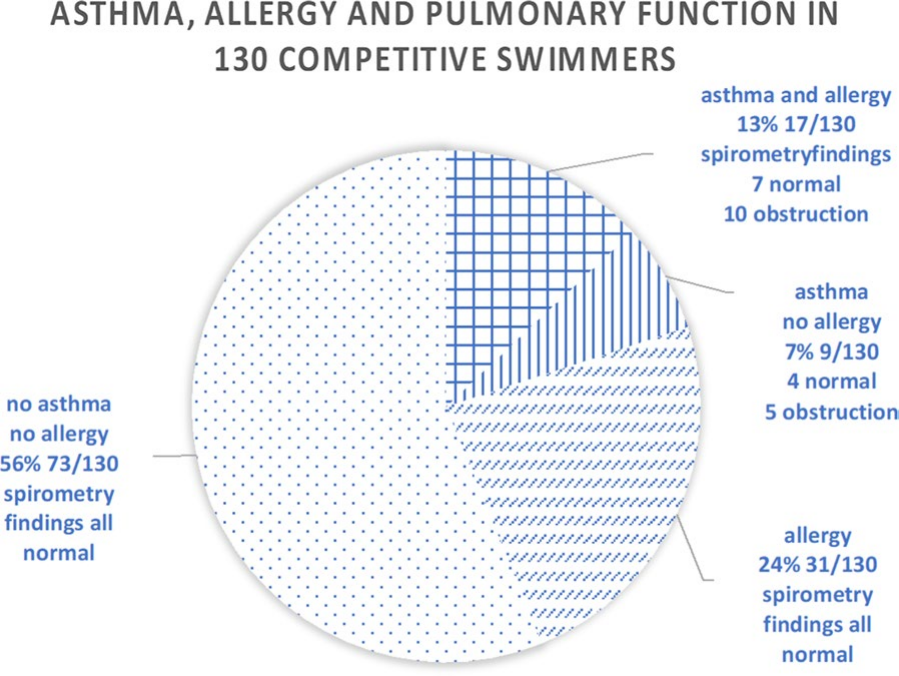
Asthma, allergies and pulmonary function findings in 130 highly trained competitive swimmers

Spirometry testing showed that the competitive swimmers had a baseline FEV_1_ and FVC mean of approximately 110% of predicted, and airway obstruction was found in 12% of the studied swimmers (Table 2). All obstruction findings were found in swimmers with physician-diagnosed asthma (Fig. 5). Surprisingly, almost all, except for two, of the swimmers with an obstruction finding, were males.

**Table 2:** Spirometry findings in 130 competitive swimmers

	All swimmers N = 130	Female swimmers N = 58	Male swimmers N = 72
FVC mean (SD)	112% (12) of predicted	111% (13) of predicted	112% (12) of predicted
FEV1 mean (SD)	108)% (13of predicted	110% (13) of predicted	106% (12) of predicted
FEV1/FVC mean (SD)	97% (9) of predicted	99% (8) of predicted	95% (9) of predicted
Airway obstruction n/N (%)	15/130 (12%)	3/58	12/72
Asthma n/N (%)	25/130 (19%)	11/58	14/72

FEV_1_: Forced expiratory volume in one second (liters per second)

FEV_1_/FVC: Ratio of Forced expiratory volume in one second and forced vital capacity (%)

FVC: Forced vital capacity (liters)

SD: Standard deviation

N: Number of subjects

n/N: Number of subjects concerned out of the whole population number of subjects

Spirometry findings, respiratory symptoms and the physician-prescribed medication were studied together (Table 3). Five of the swimmers with asthma and airway obstruction reported no medication and all of them were males.

**Table 3:** Spirometry findings, medication classified by ATC (www.whocc.no/atc_ddd_index/) and reported respiratory symptoms in 130 competitive swimmers that qualified for National Swimming Championships. The population was grouped according to physician-diagnosed asthma and allergy, physician-diagnosed asthma without allergy, physician-diagnosed allergy and no asthma and no allergy

Swimmers with physician diagnosed	Spirometry finding: Normal Obstruction §		Medication ATC classification (number of swimmers received prescription)	Respiratory symptoms
Asthma and allergy N = 17 §	10	7	∘ Respiratory system nasal preparations R01AD11 (1) ∘ R01BA52 (1) ∘ * Respiratory system drugs for obstructive airway diseases, adrenergics, inhalants R03AC02 (7) * R03AK06 (4) * R03AC03 (2) * R03AC13 (1) * R03AK07 (2) * R03BA02 (4) * R03BA05 (1) * R03BA05 (1) * × Respiratory system antihistamines for systemic use, aminoalkyl ethers R06AE07 (1) × R06AE09 (1) × R06AX22 (2) × ⦁ Sensory organs ophthalmological drugs decongestants and antiallergics S01GX01 (1) ⦁	12 out of 17
Asthma	6	3	* Respiratory system drugs for obstructive airway diseases, adrenergics, inhalants R03AC02 (4) * R03AC13 (1) * R03AK06 (2) * R03AK07 (3) * R03BA02 (1) * R03BA05 (1) * R03BA05 (1) * R03DC03 (1) * ⦁ Sensory organs ophthalmological drugs decongestants and antiallergics S01GX01 (1) ⦁	7 out of 9
Allergy N = 31	31	0	× Respiratory system antihistamines for systemic use, aminoalkyl ethers R06AE09 (1) × R06AE07 (1) × ⦁ Sensory organs ophthalmological drugs decongestants and antiallergics S01GX08 (1) ⦁	18 out of 31
No asthma and no allergy N = 73	73	0	–	9 out of 73

^§^Five swimmers with asthma and airway obstruction did not report medication usage

This study showed no differences between the training habits in swimmers using physician-prescribed medication and swimmers using no medication.

## Discussion

### Different populations

The majority of swimmers did not report any respiratory symptoms while swimming (Fig. 1) including those with physician-diagnosed asthma and allergy. During vigorous exercise, respiratory symptoms are typically observed in 90% of asthmatics. In this study, among swimmers in swimming halls with physician-diagnosed asthma, the prevalence of reported respiratory symptoms while swimming was approximately one-third of that. This confirms the previously reported findings demonstrating the low asthmogenity of swimming [10–13].

Comparisons among different activity groups being competitive, fitness and occasional swimmers showed that the competitive swimmers reported respiratory symptoms three times more often than the fitness and occasional swimmers (Fig. 1). All activity groups were swimming in the same environment of a swimming hall and were exposed to similar environmental conditions.

The subjects´ medical histories showed that those, who had physician-diagnosed allergy or had neither asthma nor allergy, had a significantly higher risk for respiratory symptoms than if they were competitive swimmers in comparison to other activity groups such as fitness swimmers and occasional swimmers (Table 2). This finding suggests that there are special factors in competitive swimming that cause reporting of respiratory symptoms. Those special factors might connect to physical exercise strain, i.e., training intensity, a water environment and being immersed.

### Time and distance for exposure

The present study showed that the length of stay under 90 min at the swimming pool area or having a swimming distance under 2000 m did not associate with reported respiratory symptoms. Typically, competitive swimmers spent time in the swimming pool area for more than 90 min and the swimming distance was far more than 2000 m (Table 1). Thus, these findings are consistent with the study of Dropnik et al. [20], who suggested that competitive swimmers are exposed to swimming hall disinfection by- products more than other activity groups in swimming halls due to the higher pulmonary ventilation during the time spent in the swimming pools. This finding of the higher prevalence of respiratory symptoms in competitive swimmers is consistent with a previous study [21]. Therefore, the exposure of competitive swimmers to the airborne trichloroamine in swimming halls may be increased by the greater amount of distance swum, and the time spent in the swimming pool area as well as the higher intensity of the physical exercise and higher ventilation rate. These factors may play a role in the increased prevalence of respiratory symptoms in competitive swimmers in comparison to other activity groups in swimming halls.

The maintenance of swimming halls, as a whole, meets the high requirements of optimal ventilation, hygiene, moisture and temperature controlling. A previous study showed that the airborne trichloroamine levels were low in swimming halls and followed the EU standards [22, 30]. Reporting of respiratory symptoms decreased after the renovation of swimming halls in Finland [31]. These findings suggest that there might be structural factors in swimming halls affecting respiratory symptoms. This suggests that renewal and good maintenance of the swimming halls may play a role in reported respiratory symptoms in swimming halls. For example, in moisture-damaged buildings toxic substances in indoor air may cause airway infections, sinusitis and bronchitis and affect respiratory symptoms. In addition, a previous study showed that the mold exposure in schools associates with elevated mold-specific IgG levels and sinusitis in teachers [32]. This study`s results are similarities with reported respiratory symptoms previously found in competitive swimmers [21]. In the previous study, sinusitis was a significant risk factor for reported respiratory symptoms in competitive swimmers [21]. However, in the studied swimming halls, no significant amounts of microbes were found [30].

There is a need for a comprehensive scientific discussion for suggestions to optimize the exposure time in the swimming hall environment for competitive swimmers. The results of this study present this need for an additional consideration concerning the medical history and combined effects of competitive swimming and water environment-specific testing for identifying the underlying risk factors for respiratory symptoms and pulmonary disease.

### Pulmonary function findings concerning asthma and allergy with prescribed medication and symptoms

Spirometry at baseline showed that the FVC in both female and male competitive swimmers was approximately 110% of predicted, which is suggested to be a typical finding in highly fit elite competitive swimmers [3, 22, 33–35]. In the present study, the body composition of competitive swimmers, such as BMI, height and weight, was consistent with those reported in elite swimmers [36].

Previous studies suggest that voluminous swimming training during childhood and adolescence stimulates lung tissue growth [37]. It is notable that when typical competitive swimmers` baseline spirometry result levels are 110% of predicted, the finding of 100% of predicted in an elite swimmer may mean that the value is a significantly lowered one for that individual. Thus, a “normal” spirometry finding in a competitive swimmer may give a misleading statement about the condition of a competitive swimmer`s pulmonary function.

The prevalence of airway obstruction findings (12%) was surprisingly high, especially because airway obstruction was mainly found in male swimmers with asthma, who did not report any respiratory symptoms during swimming. Asthmatic male swimmers had more often childhood asthma, whereas in female swimmers, asthma was diagnosed during adolescence. It may be possible that in asthmatic males, whose previous asthma was in a remission phase, had a reduction in their asthma medication usage. Some of the swimmers with airway obstruction did not report medication despite an asthma diagnosis. However, worsening of pulmonary function in asthmatic males may develop slowly without notice and airway obstruction may exist, because asthmatic male swimmers remain asymptomatic while swimming [22]. The opposite situation in female swimmers may occur. When asthmatic females sense respiratory symptoms while swimming, they will seek for medical treatment and take physician-prescribed medication. Furthermore, sensing respiratory symptoms while swimming may cause a female swimmer to avoid developing an airway obstruction. An airway obstruction finding did not associate with reported respiratory symptoms. However, in previous studies, the lowered FEV_1_/FVC associated with reported respiratory symptoms in competitive swimmers [22, 38]. The medication data were collected from the 130 competitive swimmers, who qualified for the National Championships, participated in the pulmonary function testing and the questionnaire survey. The reported physician-prescribed medication is shown in Table 3. These findings of spirometry and the questionnaire study may suggest, that the medication was mainly well balanced in asthmatic female swimmers, and a special attention on the testing and medication on asymptomatic male swimmers with asthma may be required.

## Conclusions

Swimming hall exposure, as the length of time spent at the pool area or the swimming distance, did not associate with the respiratory symptoms until swimmers exceeded 90 min or 2000 m. Typically, during competitive swimming training, the exposures are higher. Among the activity groups in swimming halls, competitive swimmers reported three times more respiratory symptoms than the other groups.

Pulmonary function findings in competitive swimmers with a 12% prevalence of airway obstruction were surprisingly high. Therefore, attention should be paid on competitive swimmers´ pulmonary health and tested through spirometry regularly to make sure that the medication is sufficient especially in those with a sensitivity of asthma and allergy.

## Data Availability

Data is not available due to the datasets generated and analyzed during the current study are not publicly available due to the regulations of ethical committee statement but are available from corresponding author upon a reasonable request. However, the raw data availability is not possible to be provided to those not involved with the study. This is stipulated by the ethical committee of Helsinki and Uusimaa hospital district, which approved the testing protocol. Permission to deliver study data to non-study personnel requires a new application for the ethical committee and new approval to deliver raw data outside the corresponding study personnel.

## Abbreviations

ATS: American Thoracic Society
ERS: European Respiratory Society
FEV_1_: Forced expiratory volume in one second, liters (l
FEV_1_/FVC: Ratio of forced expiratory volume in one second and forced vital capacity (%)
FVC: Forced vital capacity, liters (l)
N: Number of subjects
n/N: Number of subjects concerned out of the whole population
PEF: Peak expiratory flow liters per second (l·sec^−1^)
MEF_75_: Maximum expiratory flow rate at 75% of vital capacity, liters per second (l·sec^−1^)
MEF_50_: Maximum expiratory flow rate at 50% of vital capacity, liters per second (l·sec^−1^)
SD: Standard deviation
TLC: Total lung capacity, liters (l)
OR: Odds ratio
